# Scorpion Species with Smaller Body Sizes and Narrower Chelae Have the Highest Venom Potency

**DOI:** 10.3390/toxins14030219

**Published:** 2022-03-17

**Authors:** Alannah Forde, Adam Jacobsen, Michel M. Dugon, Kevin Healy

**Affiliations:** 1 Venom Systems & Proteomics Lab, School of Natural Sciences, Ryan Institute, National University of Ireland Galway, H91 TK33 Galway, Ireland; a.forde18@nuigalway.ie (A.F.); a.jacobsen2@nuigalway.ie (A.J.); michel.dugon@nuigalway.ie (M.M.D.); 2 Macroecology Lab, School of Natural Sciences, Ryan Institute, National University of Ireland Galway, H91 TK33 Galway, Ireland

**Keywords:** venom, scorpions, LD_50_, potency, body size, chela morphology, telson morphology, phylogenetic comparative analyses, defense mechanisms, evolutionary trade-off

## Abstract

Scorpionism is a global health concern, with an estimation of over one million annual envenomation cases. Despite this, little is known regarding the drivers of scorpion venom potency. One widely held view is that smaller scorpions with less-developed chelae possess the most potent venoms. While this perception is often used as a guide for medical intervention, it has yet to be tested in a formal comparative framework. Here, we use a phylogenetic comparative analysis of 36 scorpion species to test whether scorpion venom potency, as measured using LD_50_, is related to scorpion body size and morphology. We found a positive relationship between LD_50_ and scorpion total length, supporting the perception that smaller scorpions possess more potent venoms. We also found that, independent of body size, scorpion species with long narrow chelae have higher venom potencies compared to species with more robust chelae. These results not only support the general perception of scorpion morphology and potency, but also the presence of an ecology trade-off with scorpions either selected for well-developed chelae or more potent venoms. Testing the patterns of venom variations in scorpions aids both our ecological understanding and our ability to address the global health burden of scorpionism.

## 1. Introduction

Envenomation, resulting from a scorpion sting, referred to as scorpionism [1], is a major global public concern affecting Central America, South America, North Africa, the Middle East and West Asia [2]. Approximately 1.2 million scorpion stings are estimated to occur throughout the world each year, 3250 of which result in death [3,4]. Despite the global burden of scorpionism, which is likely to be higher due to unreported envenomation events [5,6], surprisingly little is understood regarding the ecology and evolution of scorpions and their venom. Such a gap in understanding and data can lead to inaccurate treatment of scorpion envenomation, through misidentification of species [7] or the misuse of antivenoms [8]. Furthermore, understanding the ecology and evolution of venoms can aid in predicting envenomation risk and its treatment, as demonstrated by the efforts related to snakebites [9]. Ecological factors have been linked to severe scorpionism, including climatic variations, venom metering and the overall morphology of the scorpion [10,11]. Understanding the ecological and evolutionary drivers of scorpion venom potency not only contributes to our fundamental understanding of these species, but also to our ability to address the global burden of scorpionism.

The concept that larger scorpions have less potent venom compared to smaller scorpions is widely expressed in the media, and has been popularized by numerous sources, such as in the Indiana Jones movie, where the main protagonist explicitly refers to this by saying “When it comes to scorpions, the bigger the better” [12]. Apart from its use in popular fiction, the concept of relying on the size of the morphological characteristics of scorpions is used as a general medical guidance in many countries where scorpionism is an issue [13,14], as most scorpions are difficult to identify at the species level by non-experts [14,15,16,17]. Support for this general concept can be found in the high median lethal dosage values (LD_50_), indicating venoms of lower potency, reported for larger species of scorpions, such as *Hadrurus arizonensis* and *Heterometrus laoticus* [18,19,20,21]. However, such general rules should be used with caution. For example, in Morocco, black scorpions are often considered as the most lethal species [22], despite evidence that the color of scorpions does not relate to lethality, and is usually determined by habitat [23]. However, unlike color, body size may have a clearer potential mechanistic relationship with venom potency.

Scorpions possess the following two main means to capture prey and dissuade predators: their venomous stinging apparatus and their pedipalps [24]. While present in all scorpions, their use varies across species, following a trade-off pattern [16]. Species that primarily rely on venom, such as many species within Buthidae, often have long slender chelae [5,24], while species with more powerful chelae, such as *Opistopthalmus glabrifrons*, typically have comparatively less-developed stinging apparatus, with a smaller metasoma, or less potent venom, as found by Lourenço [15]. From an evolutionary perspective, such a pattern may emerge, as larger species that rely more on mechanical prey capture or that avoid predators may have fewer selection pressures on the potency of their venoms. Interestingly, not only has size been used as a medical indicator in envenomation cases [25], the size of the pedipalps has been used to determine the lethality of scorpions [24,25]. However, despite popular support for these patterns, they have yet to be tested using phylogenetic comparative methods.

While phylogenetic comparative methods are a relatively recent approach to understanding venom variation [26], studies measuring venom potencies have a rich history, with numerous standardized measures of potencies, such as LD_50_, available across species spanning the scorpion phylogeny. Here, we use this rich history to collate LD_50_ potency values [20,27,28,29,30,31,32,33,34,35,36,37,38,39,40,41,42,43,44] and morphology measures [14,19,35,45,46,47,48,49,50,51,52,53,54,55,56,57,58,59,60,61,62,63,64,65,66,67,68,69,70,71,72,73,74,75,76,77,78,79,80,81,82,83] from the literature to test the commonly held belief that larger scorpions, with more powerful chelae, are comparatively less potent than smaller species. We predict that smaller species, with narrower chelae and larger telsons, will have lower LD_50_ values, indicating higher venom potency.

## 2. Results

Our dataset consisted of 36 scorpion species with 62 measures of LD_50_, with all the measures that met our criterion for inclusion using mammalian Mus musculus models (S1). The lethality of the species within the dataset ranges from 0.16 mg/kg^−1^ for the deathstalker *Leiurus quinquestriatus* to 1800 mg/kg^−1^ for the rock scorpion *Hadogenes granulatus*. The median body size was 70 mm, ranging from 40.5 mm for the bark scorpion *Scorpion Centruroides noxius* to 200 mm for *H. granulatus*. The chela ratio ranged from 0.7 to 6.2, with the most robust chelae found in the large-clawed scorpion *Scorpio maurus* and the red scorpion *Rhopalurus junceus*, with the most slender chelae found in the deathstalker *Leiurus quinquestriatus*.

**Figure 1:**
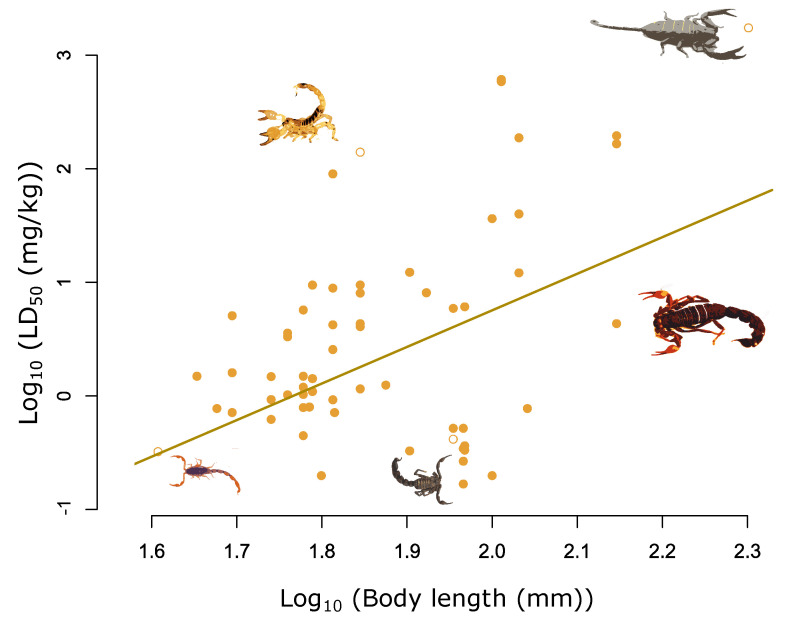
Relationship between log_10_ total length (mm) and log_10_ LD_50_ (mg/kg) for 62 measures of LD_50_ across 36 species. The fitted line highlights the significant positive relationship between log_10_ total length and log_10_ LD_50_, adjusted for the median chelae ratio value of 3.7 (β = 3.24, pMCMC < 0.001). Selected species highlighted from left to right are highlighted by the hollow yellow circles and are as follows: *Scorpion Centruroides noxius*; *Scorpio maurus*; *Androctonus crassicauda*; *Parabuthus transvaalicus*; *Hadogenes granulatus*.

We found that body length was positively correlated with LD_50_, with the larger scorpions being associated with less potent venoms (Figure 1 and Figure S1 and Table 1). The significant slope between log_10_ body length and log_10_ LD_50_ found in our analysis corresponds to a change in LD_50_ of 187 mg/kg^−1^ across the 159 mm range of body length in our dataset (Figure 1 and Figure S1 and Table 1).

**Figure 2:**
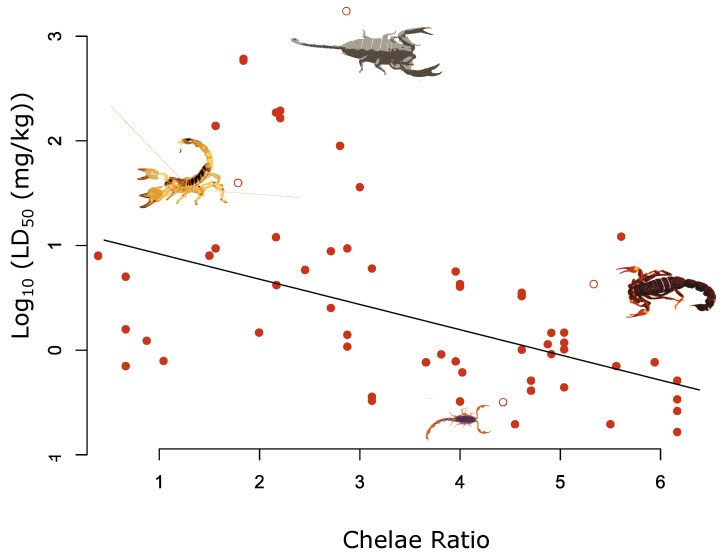
Relationship between chelae and log_10_ LD_50_ (mg/kg) for 62 measures of LD_50_ across 36 species. The fitted line highlights the significant negative relationship between the chelae ratio value and log_10_ LD_50_ adjusted for the median log_10_ total length value of 70 mm (β = −0.26, pMCMC < 0.01). Selected species are highlighted by the hollow red circles and from left to right are as follows: *Scorpio maurus*; *Hadogenes granulatus*; *Scorpion Centruroides noxius*; *Parabuthus transvaalicus*.

For the morphological traits, we found a significant negative relationship between log_10_ LD_50_ and chelae ratio, with species possessing more slender chelae being associated with lower log_10_ LD_50_ values (Figure 2 and Figure S1 and Table 1). Our results show that increasing the chelae ratio by one unit results in a 0.26 decrease in log_10_ LD_50_. Across the full range of chelae ratios in our dataset, this corresponds to approximately an order of magnitude decrease in LD_50_ (Figure 2 and Table 1).

For the telson ratio and the methods of injection for the LD_50_ test, we found no significant relationship with log_10_ LD_50_. The phylogenetic signal associated with LD_50_ was moderate to high in the analysis, with a h^2^ value of 0.60, and little variation associated with the species (Table 1).

## 3. Discussion

Here, we found strong support for the widely held perception that larger scorpions, with more robust chelae, are less potent than small species, with thin chelae. These results follow the general observations that the most potent scorpion venoms are recorded in smaller species, such as the bark scorpion *Scorpion Centruroides noxius* or the Brazilian yellow scorpion *Tityus serrulatus*. Conversely, the highest LD_50_ values, and, hence, the least potent scorpion venoms, are often found in some of the largest scorpion species. For example, in our data, the largest species, the rock scorpion *Hadogenes granulatus*, is associated with the least potent venom. Similarly, species with robust, well-developed chelae, such as the Israeli gold scorpion *Scorpio maurus*, were also associated with some of the least potent venoms. More potent venoms were found in species with narrow chelae, such as the South African thick-tail scorpion *Parabuthus transvaalicus*.

From an ecological perspective, these results support the patterns expected to arise from an evolutionary trade-off in investment between systems that can play similar defensive and predatory roles [16,84]. For prey capture, scorpions are observed to either primarily rely on mechanically capturing and subduing their prey with their pedipalps or only using them in a supportive role, with their venom primarily carrying out the function of incapacitation [85,86,87]. Such a trade-off is particularly apparent in species with wide chelae that can produce larger crushing forces, but have less-developed metasoma, such as in the rock scorpion *Hadogenes granulatus*.

Interestingly, we did not find a correlation between the shape of the telson, which houses the venom glands, and the venom potency. This highlights that, while there may be an evolutionary trade-off between chelae development and venom potency, this trade-off is not linked to the telson morphology. Instead, this suggests reduced selection in maintaining high levels of potency in larger species with robust chelae, but not in the ability to deliver venom. While snake species that no longer utilize venom for predation are found to have reduced potencies and, in some cases, are no longer able to deliver venom [88], this does not seem to be the case in scorpions. This retained ability to deliver venom in scorpion species that no longer use venom for predation likely points to the important defensive function of scorpion venoms. However, while our results do not support a trade-off between telson morphology and venom potency, other features of the scorpion delivery system may be associated with this trade-off, such as the metasoma. For example, Ref. [15] associated the thickness of the metasoma with venom potency. This may highlight the relationship between how frequently venom is used by a species and the fact that species that rely on venom for predation require more well-developed metasoma.

Our results highlight how ecological drivers can select for venom potency. However, the functional abilities of scorpion venoms are far more complex than the relatively one-dimensional measure of lethality using LD_50_. For example, it would be predicted that species with less-developed metasomas, such as the rock scorpion, may primarily retain venoms for defensive purposes, with such venoms no longer being selected for lethality, but for other attributes, such as inducing pain [89]. Conversely, while LD_50_ measures may be more appropriate to capture the functional ability of species that primarily rely on venom to incapacitate prey, other measures, such as the time taken to incapacitate, may be more ecologically relevant for future studies [90].

Further to the type of potency measure used, accounting for the species on which the venom is tested may also provide further understanding of potency variation across scorpion species. As the data used in our analysis consist of potency measures tested on mammalian models, more ecologically relevant test models that reflect each scorpion species’ diet may allow more detailed analyses of the drivers of scorpion venoms and composition to be conducted [26,91]. Studies have indicated some level of prey specificity in scorpion venom [92]; however, very few studies have tested scorpion venoms against their typical targets. The inclusion of such ecologically relevant test models would allow for the use of similar evolutionary distance metrics to those used in studies of snake venoms [26,93], to test and account for potency variation associated with prey specificity. Such models may also shed further light on the lack of a relationship found here between the route of venom injection and the potency. While it has been found, in studies of snake venoms, that the measures of LD_50_ using intravenous routes of injection are lower, the lack of such a relationship here may potentially be due to the fact that the scorpion venoms were selected to act on invertebrate prey, where the intravenous injection of venom was not selected for. However, as the potency measures used here were tested on mammalian model species, our results likely reflect the expected potency of scorpion venoms from a human medical perspective.

Our results also support the use of general scorpion morphology as a broad indicator for assessing the potential potency of a species. Such general indicators, particularly for clearly identifiable features, such as pedipalp size, may aid in envenomation cases where a species has not been identified, an issue that is common in many tropical and sub-tropical regions [94]. However, caution should be taken when using such general rules, as there is a large degree of variation in potency across the general patterns found in our analysis. For example, *Scorpio maurus* and *Androctonus crassicauda* are relatively similarly sized species, yet, despite *Androctonus crassicauda* being slightly larger, it is orders of magnitude more potent compared to *Scorpio maurus*. Hence, the use of these general indicators should be firmly based on more regional-specific information regarding the species that are likely to be associated with an envenomation event [95].

## 4. Conclusions

While potency is often the primary focus in understanding scorpionism risk, understanding the link between scorpion ecology and venom composition, and their associated envenomation symptoms [96], is likely to help in developing suitable envenomation remediation strategies. Testing fundamental patterns associated with the drivers of variation in scorpion venoms will not only allow for a clearer understanding of the ecology and evolution of venoms in scorpions, but will also provide a clearer path in understanding how to address the global issue of scorpionism.

## 5. Materials and Methods

To test our hypothesis, we collated data on venom potency and morphological features from the available literature. We performed an initial Web of Science search for scorpion potency and morphology data using the following search terms: “scorpion(s)”, “scorpion venom”, “scorpion venom potency”, “medically significant scorpions”, “lethality”, “median lethal dose”, “scorpion LD_50_”, “pedipalp measure”, “total body size”, “chela size”, “chela length”, “chela width”, “telson measure”, “telson length” and “telson width”. Further citations within key sources were also used in conjunction with the search terms.

For venom potency, we used median lethal dose (LD_50_), which was administered by intravenous (IV), subcutaneous (SC), intraperitoneal (IP) or intramuscular (IM) routes. We only included dried venom LD_50_ values, which reported the body mass of the test species and converted all units to mg of dried venom to kg of test subject.

**Figure 3:**
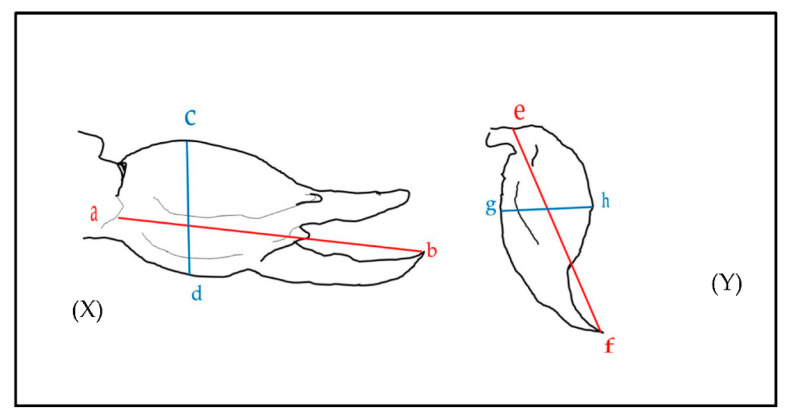
Measurements taken from diagrams and photos with scale bars. (**X**) shows measurements for the length (a to b) and width (c to d) of the chela. (**Y**) shows the length (e to f) and width (g to h) of the telson. (a) represents the distal point where the pedipalpal patella meets the tibia; (b) is the most distal point of the pedipalpal tibia; (c) and (d) are the widest dorsoventral points on the pedipalpal tibia; (e) is the posterior end of the venom vesicle; (f) is the distal end of the aculeus; (g,h) are the widest dorsoventral points of the telson.

For morphological measurements, we used scorpion total body size (mm) and the length and width (mm) of the chela and telson from reported values and from diagrams and photos where scale bars were present. For measurements for the length of the chela, we used maximum distance from the tip of the tarsus (Figure 3X(a) to the base of the tibia, where it meets the distal end of the patella (Figure 3X(b). The width of the chela was measure at the widest points of the tibia (Figure 3X(c,d). For the telson, the length was measured as the maximum distance between the base of the vesicle (e) and the distal end of the aculeus (f), and the width was measured at the widest point of the vesicle (between points g and h).

As morphological measures were not available for *Centruroides limpidus,* we used values available from *Centruroides ruana*. These were described to have identical morphological features as each other.

For the analysis, we log_10_ transformed LD_50_ and body size values, as they resulted in a more normal distribution of model residuals. As body size is expected to be related to the length and width of both the chela and telson, we used length–width ratios to give mass-independent indices of their morphology. As short thick chelae are associated with delivering higher forces compared to long thick chelae [84,97], we divided the length of each chela by its width. Hence, this chela ratio ranged from high values, representing long thin chelae, to low values, representing short thick chelae. For the telson, we also divided the telson length by its dorsoventral width to give values ranging from high values, representing narrow elongated telsons, which are expected to be associated with a less-developed telson [98], to lower values, representing more bulbus telsons, which are expected to be associated with more developed use of venom [98].

To test our hypotheses, we fitted Bayesian phylogenetic mixed models (BPMM), using the MCMCglmm package [99] in R version 4.0.4 (Team 2016). These models allow for the inclusion of multiple explanatory variables as fixed effects, and random effect terms, which can be used as variance terms [99]. We controlled for pseudoreplication, due to shared ancestry between species, by using the animal term, and for the phylogenetic relationships between scorpions from our dataset using a phylogeny from the Open Tree of Life project [100]. The animal term uses a distance matrix of the phylogenetic distance between species to control for the expected similarity in trait values [99]. We calculated the relative variance attributable to the animal term as h^2^ [101], which can be interpreted in a similar fashion to the phylogenetic lambda value. A h^2^ value close to 1 indicates a Brownian model of trait evolution, while a value close to zero indicates independence between trait values [101]. In order to include multiple measures of LD_50_ for each species in our analysis, we also used a random term for species, similar to previous comparative models of venom variation across taxonomic groups [26]. Significance for a fixed term is determined when the 95% credibility interval (CI) does not cross zero [99]; however, we also included pMCMC values, which are a Bayesian alternative to *p*-values, with significance interpreted when pMCMC < 0.05 [99].

We fitted all models using standard non-informative priors, with the burn-in, thinning and number of iterations determined to ensure effective sample sizes exceeded 1000 for all parameter estimates. We tested for convergence using the Gelman–Rubin statistic over three separate chains [102].

As we found LD_50_ and body length to have log-normal distributions, we log_10_ transformed these variables. Such a transformation also allows us to fit a power–law relationship between LD_50_ and body length of the form LD_50_ ~ (body length)^a^. Such power relationships are typically associated with scaling relationships with body size [103], and have also been found to be associated with body size and venom properties in studies of snake venom [26]. We tested for collinearity between the independent factors using a series of regressions, finding no significant correlations between them (Supplementary Figures S2 and S3). We controlled for the effect of route of injection by including it as a fixed factor (SC, IM, IV, and IP), along with the two random terms accounting for phylogenetic variation (“animal” term) and species-level variation (“species” term). In our model, we included log_10_ (LD_50_) as the response variable, with log_10_ (body length), chela ratio and telson ratio as the independent variables. All data and analysis code are available in the Supplementary Files S1–S3.

## Figures and Tables

**Table 1:** Main models testing the role of body size and morphology on LD_50_. The modes (β) and 95% credibility intervals (lower CI and upper CI) of the posterior distributions are given for all fixed and random terms in a model, with log_10_ of LD_50_ as the response variable. Fixed terms include the continuous factors log_10_ of scorpion total length, the ratio of chela length to width (chela ratio) and the ratio of telson length to width (telson ratio). Categorical fixed terms include the LD_50_ method of injection (subcutaneous (SC), intravenous (IV), intraperitoneal (IP) and intramuscular (IM)), with SC as the baseline. The random terms associated with phylogenetic relatedness (phylogeny (h^2^)), intraspecific variation (species) and residual variation (residual) are also presented. For more details on the parameters, see Materials and Methods. The model has 62 LD_50_ measures for 36 species.

	β	Lower CI	Upper CI	pMCMC
**Fixed Terms**				
Intercept	**−4.82**	**−8.40**	**−1.68**	**0.004**
Log_10_ body length (mm)	**3.24**	**1.57**	**4.99**	**<0.001**
LD_50_ method_SC_				
*IV*	−0.04	−0.35	0.29	0.81
*IP*	0.17	−0.36	0.73	0.88
*IM*	0.10	−1.05	1.35	0.52
Chela ratio	**−0.26**	**−0.40**	**−0.11**	**0.005**
Telson ratio	0.04	−0.15	0.20	0.63
**Random Terms**				
Phylogeny (h^2^)	0.60	0.25	0.87	
Species	0.01	0.00	0.46	
Residuals	0.26	0.10	0.49	

Significant values, which are highlighted in bold, are deemed to be those with 95% of the posterior estimate above or below zero.

## Data Availability

All data used in the analysis can be found in the Supplementary File S1.

## Supplementary Materials

The following supporting information can be downloaded at https://www.mdpi.com/article/10.3390/toxins14030219/s1, Figure S1: Three dimensional plot of the relationship between the two significant the independent effects of log10 transformed Body size and Chela ratio with log10 transformed LD50. (for 62 observations for 36 species); Figure S2: Relationship between Chela ratio and log10 body length demonstrating no significant relationship between the variables (Slope = −0.002, *p* = 0.89 for 62 observations for 36 species); Figure S3: Relationship between Telson ratio and log10 body length demonstrating no significant relationship between the variables (Slope = −0.01, *p* = 0.45 for 62 observations for 36 species); File S1: Dataset containing measures of LD50, morphological measures and references for the data used in the analysis; File S2: Script outlining the analysis; File S3: Phylogeny file required for comparative analysis.
