# Supplementary material for: Scorpion Species with Smaller Body Sizes and Narrower Chelae Have the Highest Venom Potency
Source: Toxins (Basel). 2022 Mar 17;14(3):219. doi: 10.3390/toxins14030219 (PMC8951363; doi:10.3390/toxins14030219)
Supplement: Supplementary file 1 [file toxins-14-00219-s001.zip › File S2_script.pdf]

Forde_et_al_2022_script

This document outlines the code used to run the analysis in Forde et al 2022.

First we need to load the packages we need for the analysis. This includes the "MCMCglmm" package which will carry out the phylogentic bayesian analysis. More information on this package can be found in the course notes (http://www2.uaem.mx/r-mirror/web/packages/MCMCglmm/MCMCglmm.pdf)

The other package we need is the "hdrcde" which we use to caluclate mode values. Next we need the "caper" package to uplaod the phylogeny and finally we need "scatterplot3d" for the 3D plot.

```
#These packages need to be install the first time it is run
#install.packages(hdrcde)
#install.packages(MCMCglmm)
#install.packages(caper)
#install.packages(scatterplot3d)

library(MCMCglmm)
library(hdrcde)
library(caper)
library(scatterplot3d)
```

Now we will upload the csv file with the data, which contains the LD50 data and the morphology data.

```
LD50_data <- read.csv("S1_data.csv",
                      sep = ",",
                      header = T)
```

Next we upload the phylogeny that matches the data using the read.tree function.

```
scorpian_phylo <- read.tree("S4_phylogeny")
```

Since we don't need all the data in the uploaded dataseet we will subset it to just the variables we are intrested in. We also need to create a colunm called 'animal' that is just a dublicate of the species colunm for MCMCglmm to match up with the phylogeny.

```
#create the animal variable
LD50_data$animal <- LD50_data$species

#only include the variables we want
LD50_size_method <- LD50_data[,c("species",
                                 "animal",
                                 "body_size_median_mm",
                                 "ld50_mg_kg",
                                 "ld50_method",
                                 "telson_length_mm",
                                 "telson_width_mm",
                                 "chela_length_mm",
                                 "chela_width_mm")]
```

We can now calculate the telson and chela ratios using the length and width data.

```
#Calculate Telson_ratio
LD50_size_method$Telson_ratio <- LD50_size_method$telson_length_mm/
                                  LD50_size_method$telson_width_mm

#Calculate Chela_ratio
LD50_size_method$Chela_ratio <- LD50_size_method$chela_length_mm/
                                LD50_size_method$chela_width_mm

#Removing NA
LD50_size_method_no_na <- na.omit(LD50_size_method)


#Set it so that sc is the baseline for the levels
LD50_size_method_no_na$ld50_method <- factor(LD50_size_method_no_na$ld50_method,
                                             levels = c("sc","iv","im","ip"))
```

We will use a Baysian modelling approach to incorporate the phylogeny. To do this we need a prior. Here we set it to be uniformative where we have no prior expectation of the vaules of any of the parameters in the model.

```
prior <- list(R = list(V = 1, nu=0.002),
            G = list(G1=list(V = 1, nu=0.002, alpha.mu= 0, alpha.V= 10^3),
                     G2=list(V = 1, nu=0.002, alpha.mu= 0, alpha.V= 10^3)
                     ))
```

Now we run the model. We use MCMCglmm to run a multiple regression with log10 of LD50 as the response variable and log10 of body mass, the methods of venom injection and both the telson and chela ratios as explanitory variables. To control for multple measures per species a random effect terms "species" was included. To account for non-independence due to common descent across species we include the phylogeny using the "animal" term.

We run the model for 240000 iternation with a burn-in of 40000 and a thinning of 100. We ran two independent chains to ensure convergance on the parameter values.

```
Mod_1 <- MCMCglmm(log10(ld50_mg_kg) ~ log10(body_size_median_mm)
                                      + ld50_method
                                      + Telson_ratio
                                      + Chela_ratio,
                       random = ~ animal + species,
                       pedigree = scorpian_phylo$phy,
                       data = LD50_size_method_no_na,
                       family=c("gaussian"),
                       nitt = 240000,
                       thin = 100,
                       burnin = 40000,
                       prior = prior,
                       verbose =  F)

Mod_2 <- MCMCglmm(log10(ld50_mg_kg) ~ log10(body_size_median_mm)
                                      + ld50_method
                                      + Telson_ratio
                                      + Chela_ratio,
```

```
                        random = ~ animal + species,
                        pedigree = scorpian_phylo$phy,
                        data = LD50_size_method_no_na,
                        family=c("gaussian"),
                        nitt = 240000,
                        thin = 100,
                        burnin = 40000,
                        prior = prior,
                        verbose =  F)

summary(Mod_1)
```

```
##
##  Iterations = 40001:239901
##  Thinning interval  = 100
##  Sample size  = 2000
##
##  DIC: 109.7605
##
##  G-structure:  ~animal
##
##        post.mean  l-95% CI u-95% CI eff.samp
## animal    0.2031 2.071e-09   0.5523     2000
##
##                ~species
##
##         post.mean  l-95% CI u-95% CI eff.samp
## species     0.206 1.729e-06   0.5567     2000
##
##  R-structure:  ~units
##
##       post.mean l-95% CI u-95% CI eff.samp
## units    0.2248    0.108   0.3741     2000
##
##  Location effects: log10(ld50_mg_kg) ~ log10(body_size_median_mm) + ld50_method + Telson_ratio + Che
##
##                            post.mean l-95% CI u-95% CI eff.samp  pMCMC
## (Intercept)                 -4.81473 -8.39583 -1.68313     2000  0.004 **
## log10(body_size_median_mm)   3.24199  1.56797  4.99058     2000 <5e-04 ***
## ld50_methodiv               -0.03677 -0.34477  0.28561     2000  0.805
## ld50_methodim                0.09600 -1.04712  1.34920     2000  0.875
## ld50_methodip                0.17188 -0.36018  0.72700     2000  0.519
## Telson_ratio                 0.04141 -0.15318  0.20268     2000  0.627
## Chela_ratio                 -0.25543 -0.40268 -0.11077     2000  0.005 **
## ---
## Signif. codes:  0 '***' 0.001 '**' 0.01 '*' 0.05 '.' 0.1 ' ' 1
```

We then test whether the chains converged using gelman.diag. If the values depart from 1 they indicate that convernganace has not been met based on a potential scale reduction factor (see ?gelman.diag) for more details.

```
chain_list <- mcmc.list(Mod_1$Sol, Mod_2$Sol)
gelman.diag(chain_list)
```

```
## Potential scale reduction factors:
##
##                              Point est. Upper C.I.
## (Intercept)                           1       1.01
## log10(body_size_median_mm)            1       1.01
## ld50_methodiv                         1       1.00
## ld50_methodim                         1       1.01
## ld50_methodip                         1       1.01
## Telson_ratio                          1       1.00
## Chela_ratio                           1       1.00
##
## Multivariate psrf
##
## 1.01
```

We can now plot the results.These are the "raw" graphs, with illustrations added outside of R using inkscape. Note also that as we are plotting 2D graphs we plot the graph at the median value of the other significant independent factor.

First we plot body body size

```
plot(log10(ld50_mg_kg)~ log10(body_size_median_mm),
           data = LD50_size_method_no_na,
     pch = 16,
     bty = "n",
     ylab = "log10 LD50",
     xlab = "log10 body length")

abline(hdr(Mod_1$Sol[,1])$mode +
         median(LD50_size_method_no_na$Chela_ratio)*
         hdr(Mod_1$Sol[,7])$mode,
       hdr(Mod_1$Sol[,2])$mode)
```

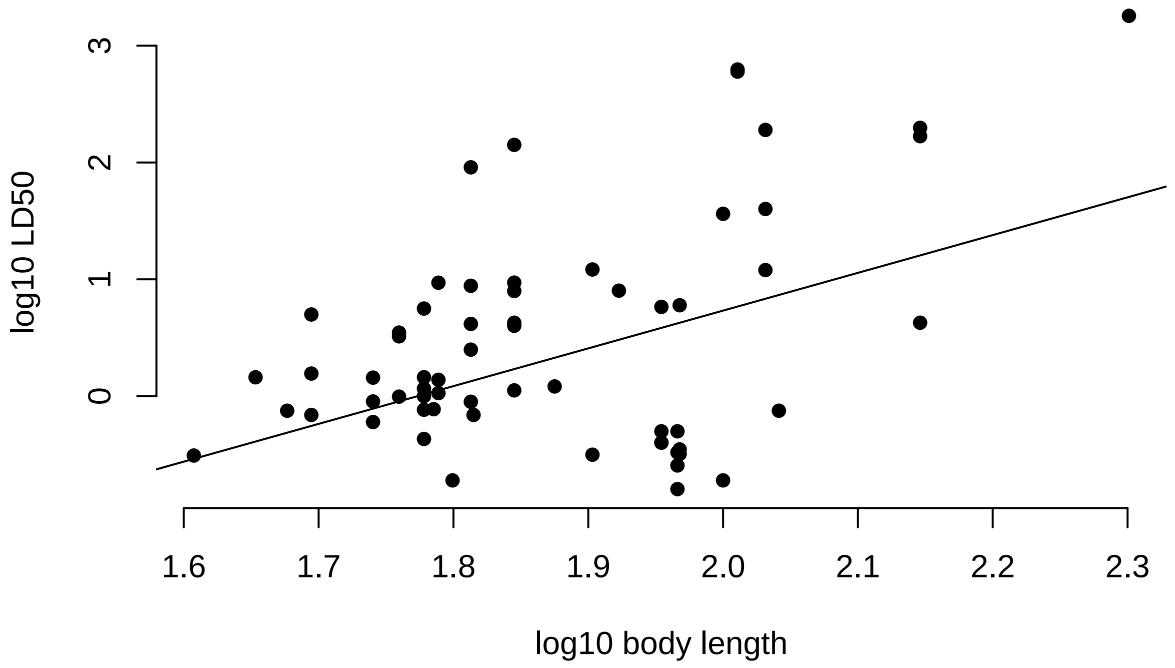

and next the Chela ratio

```
plot(log10(ld50_mg_kg)~ Chela_ratio,
           data = LD50_size_method_no_na,
     pch = 16,
     bty = "n",
     ylab = "log10 LD50",
     xlab = "Chela ratio")

abline(hdr(Mod_1$Sol[,1])$mode +
         median(log10(LD50_size_method_no_na$body_size_median_mm))*
         hdr(Mod_1$Sol[,2])$mode,
       hdr(Mod_1$Sol[,7])$mode)
```

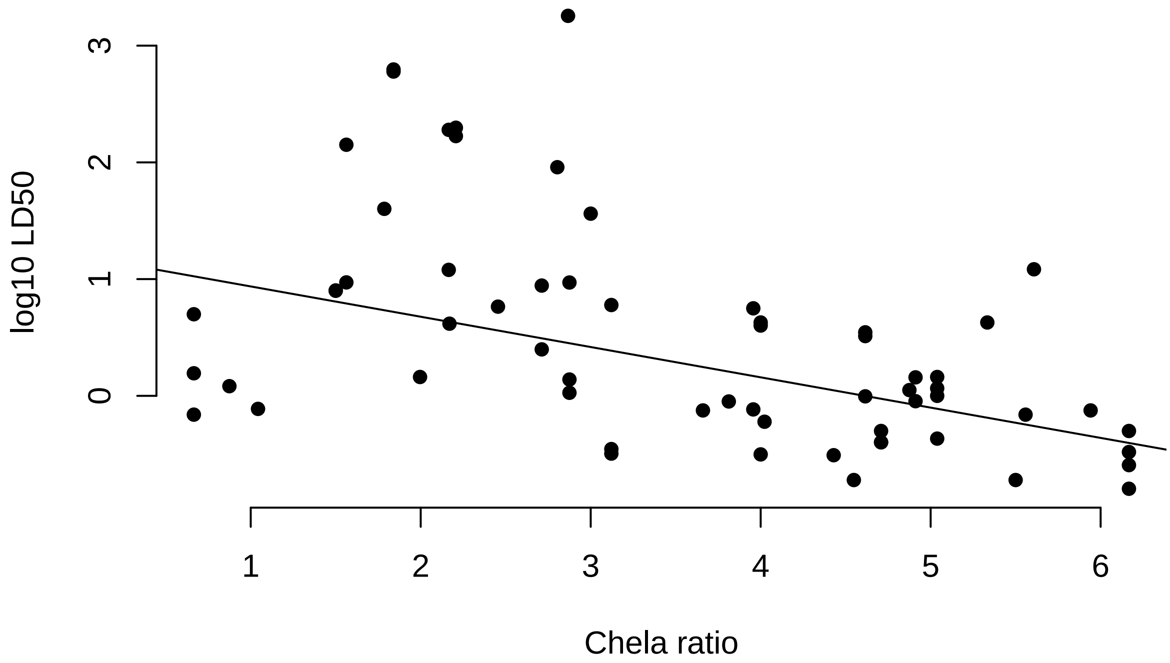

We can also plot the 3D graph with both variables included to visualise the joint distribution of the model we fit.

```
td_data <- data.frame(
                      Log10_Body_length = log10(LD50_size_method_no_na$body_size_median_mm),
                      Chela_ratio = LD50_size_method_no_na$Chela_ratio,
                      Log10_LD50 = log10(LD50_size_method_no_na$ld50_mg_kg))

s3d <- scatterplot3d(td_data,
              pch = 16,
              angle=60,
              grid=TRUE)

my.lm <- lm(td_data$Log10_LD50 ~ td_data$Log10_Body_length
            + td_data$Chela_ratio)
s3d$plane3d(my.lm)
```

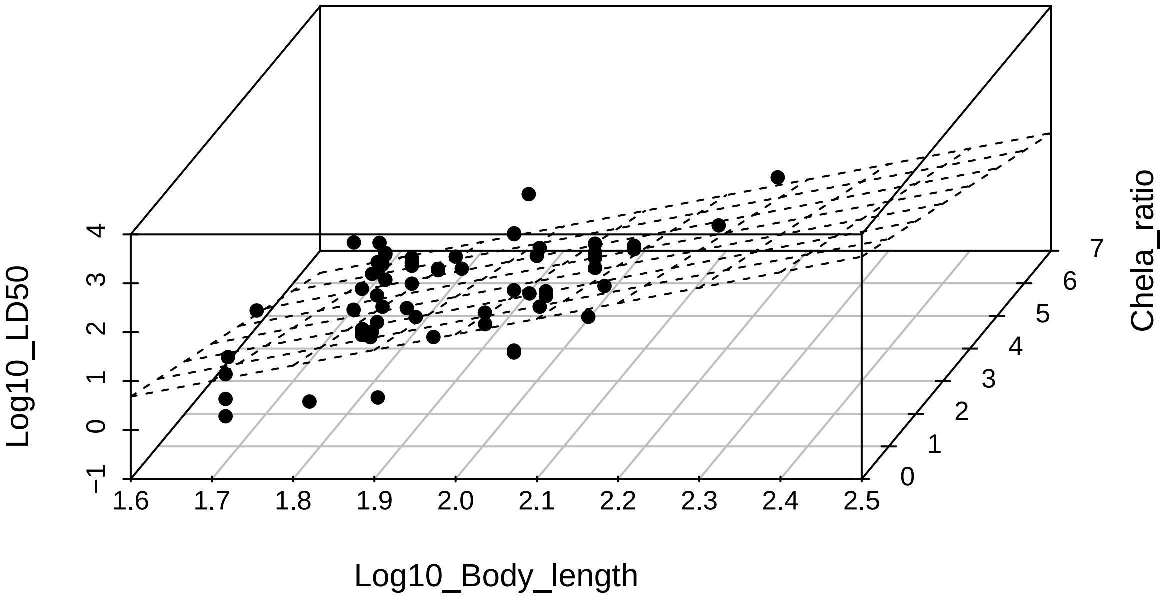

We can also test that the ratios and body size are not correlated.

```
#GLM

body_chela_mod <- glm(log10(body_size_median_mm) ~ Chela_ratio,
                       data = LD50_size_method_no_na,
                       family=c("gaussian"))

summary(body_chela_mod)
```

```
##
## Call:
## glm(formula = log10(body_size_median_mm) ~ Chela_ratio, family = c("gaussian"),
##     data = LD50_size_method_no_na)
##
## Deviance Residuals:
##      Min        1Q    Median        3Q       Max
## -0.26495  -0.09329  -0.03182   0.09569   0.42617
##
## Coefficients:
##              Estimate Std. Error t value Pr(>|t|)
## (Intercept)  1.879379   0.042716  43.997   <2e-16 ***
## Chela_ratio -0.001575   0.011051  -0.143    0.887
## ---
## Signif. codes:  0 '***' 0.001 '**' 0.01 '*' 0.05 '.' 0.1 ' ' 1
##
```

```
## (Dispersion parameter for gaussian family taken to be 0.01885519)
##
##     Null deviance: 1.1317  on 61  degrees of freedom
## Residual deviance: 1.1313  on 60  degrees of freedom
## AIC: -66.285
##
## Number of Fisher Scoring iterations: 2
```

and plot it.

```
plot(log10(body_size_median_mm) ~ Chela_ratio,
           data = LD50_size_method_no_na,
     pch = 16,
     bty = "n",
     ylab = "log10 body length (mm)",
     xlab = "Chela ratio")

abline(body_chela_mod)
```

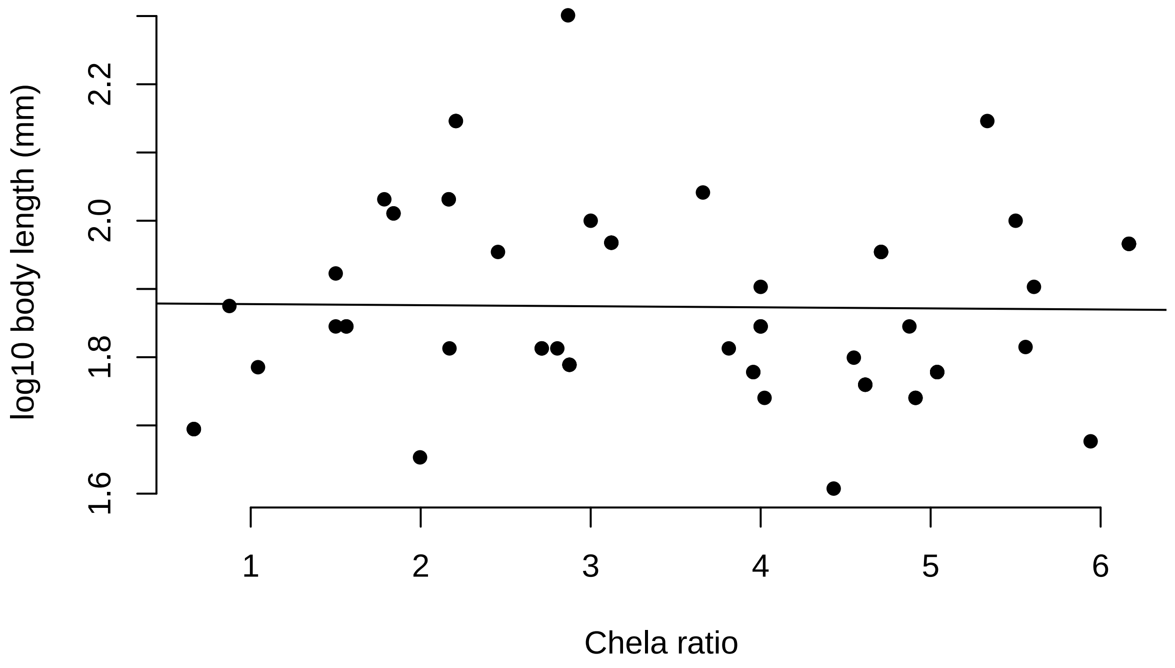

Next we test that the telson ratio and body size are not correlated.

```
#GLM

body_telson_mod <- glm(log10(body_size_median_mm) ~ Telson_ratio,
                       data = LD50_size_method_no_na,
                       family=c("gaussian"))
```

```r
summary(body_telson_mod)
```

```
##
## Call:
## glm(formula = log10(body_size_median_mm) ~ Telson_ratio, family = c("gaussian"),
##     data = LD50_size_method_no_na)
##
## Deviance Residuals:
##      Min        1Q    Median        3Q       Max
## -0.26630  -0.08738  -0.03074   0.09231   0.41957
##
## Coefficients:
##               Estimate Std. Error t value Pr(>|t|)
## (Intercept)   1.903213   0.042318  44.974   <2e-16 ***
## Telson_ratio -0.009516   0.012495  -0.762    0.449
## ---
## Signif. codes:  0 '***' 0.001 '**' 0.01 '*' 0.05 '.' 0.1 ' ' 1
##
## (Dispersion parameter for gaussian family taken to be 0.018681)
##
##     Null deviance: 1.1317  on 61  degrees of freedom
## Residual deviance: 1.1209  on 60  degrees of freedom
## AIC: -66.86
##
## Number of Fisher Scoring iterations: 2
```

and plot that

```r
plot(log10(body_size_median_mm) ~ Telson_ratio,
           data = LD50_size_method_no_na,
     pch = 16,
     bty = "n",
     ylab = "log10 body length (mm)",
     xlab = "Telson ratio")

abline(body_telson_mod)
```

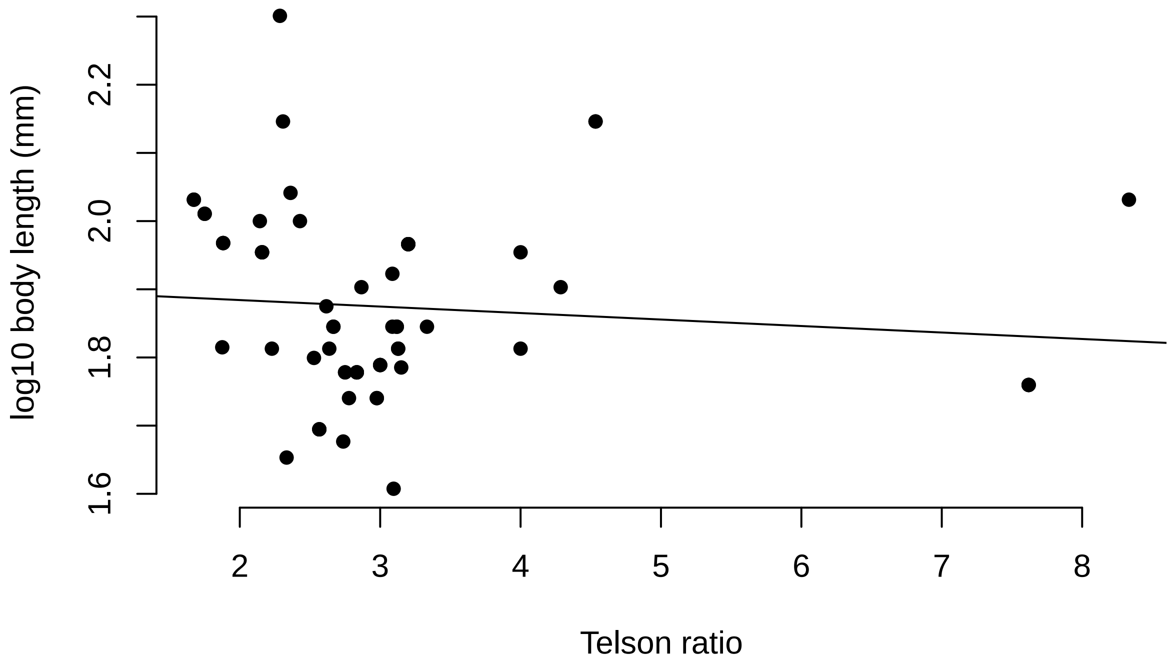

log10 body length (mm)
1.6
1.8
2.0
2.2
2
3
4
5
6
7
8
Telson ratio
