# Supplementary material for: Scorpion Species with Smaller Body Sizes and Narrower Chelae Have the Highest Venom Potency
Source: Toxins (Basel). 2022 Mar 17;14(3):219. doi: 10.3390/toxins14030219 (PMC8951363; doi:10.3390/toxins14030219)
Supplement: Supplementary file 1 [file toxins-14-00219-s001.zip › toxins-1605801 supplementary.pdf]

toxins

MDPI

Article

Scorpion Species with Smaller Body Sizes and Narrower Chelae Have the Highest Venom Potency

Alannah Forde, Adam Jacobsen, Michel M. Dugon and Kevin Healy

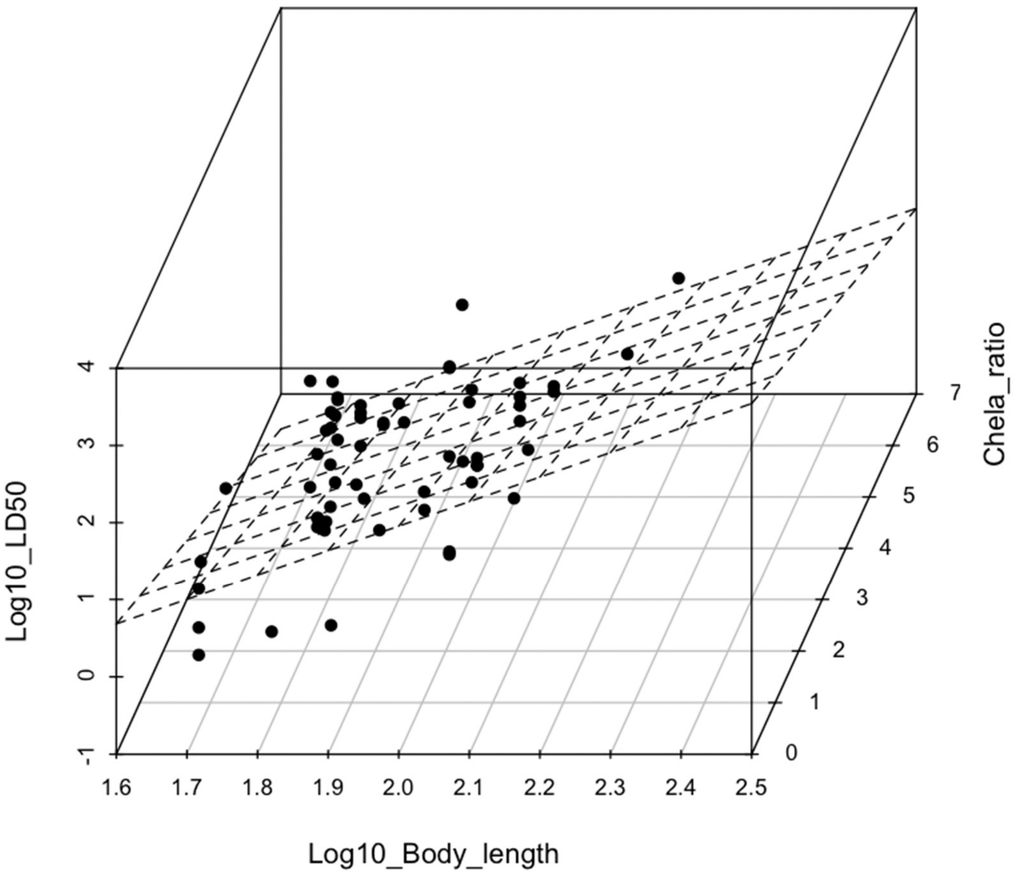

Figure S1. Three dimensional plot of the relationship between the two significant the independent effects of log_{10} transformed Body size and Chela ratio with log_{10} transformed LD_{50}. (for 62 observations for 36 species).

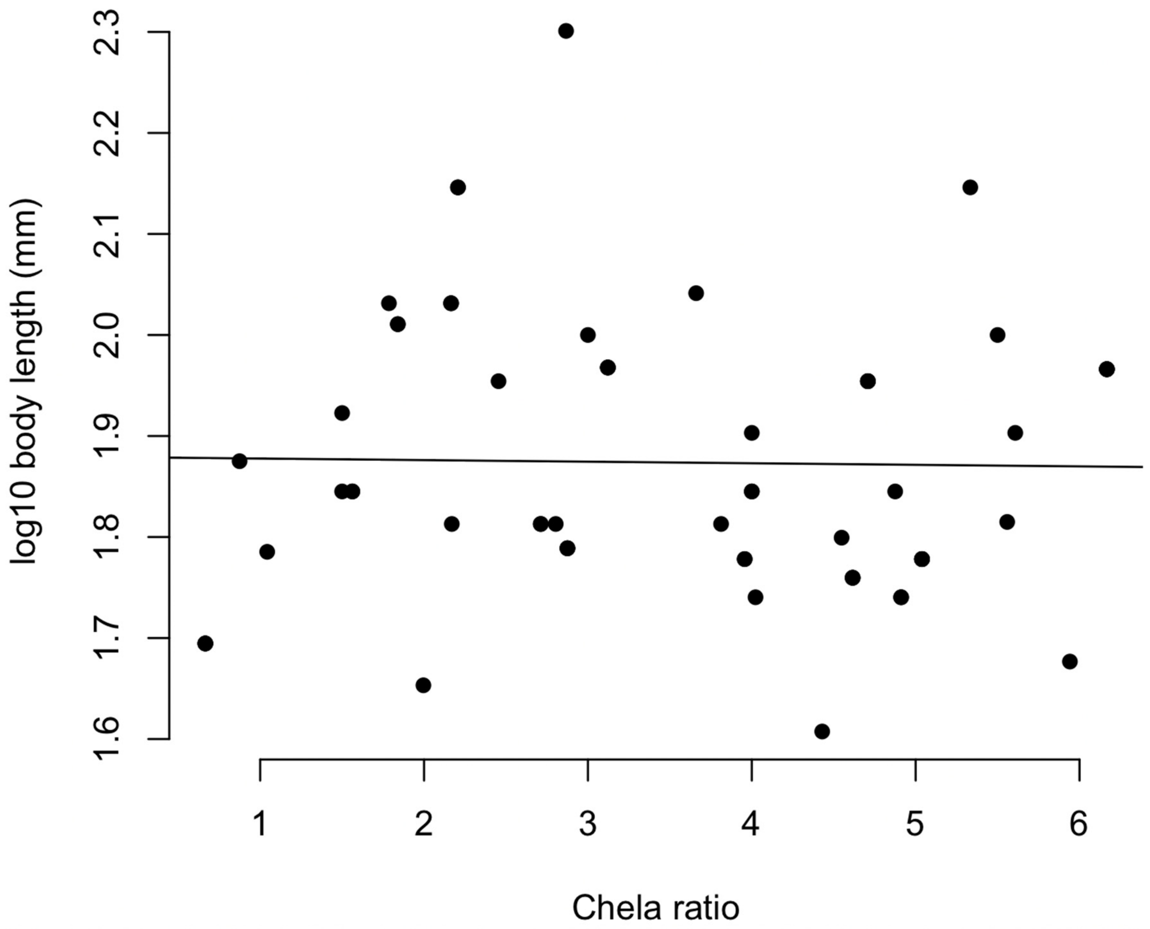

Figure S2. Relationship between Chela ratio and $\log_{10}$ body length demonstrating no significant relationship between the variables (Slope = -0.002, p = 0.89 for 62 observations for 36 species).

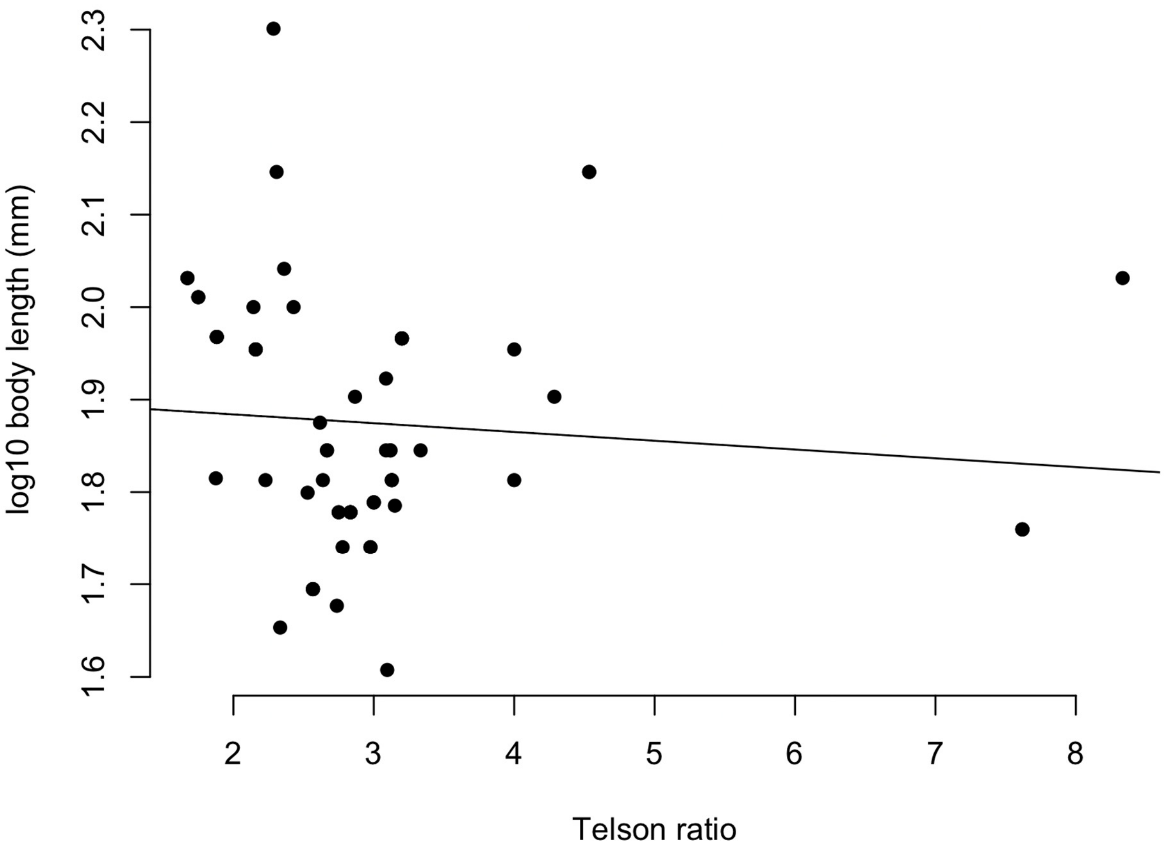

Figure S3. Relationship between Telson ratio and log_{10} body length demonstrating no significant relationship between the variables (Slope = -0.01, p = 0.45 for 62 observations for 36 species).
